# Nuclear localization of Annexin A7 during murine brain development

**DOI:** 10.1186/1471-2202-6-25

**Published:** 2005-04-10

**Authors:** Michaela Rick, Soraya I Ramos Garrido, Claudia Herr, Dietmar R Thal, Angelika A Noegel, Christoph S Clemen

**Affiliations:** 1 Center for Biochemistry, Institute of Biochemistry I, Medical Faculty and Center for Molecular Medicine Cologne, University of Cologne, Joseph-Stelzmann-Str. 52, 50931 Köln, Germany; 2 Institute of Neuropathology, University Hospital Bonn, Sigmund-Freud Str. 25, 53105 Bonn, Germany

## Abstract

**Background:**

Annexin A7 is a member of the annexin protein family, which is characterized by its ability to interact with phospholipids in the presence of Ca^2+^-ions and which is thought to function in Ca^2+^-homeostasis. Results from mutant mice showed altered Ca^2+^-wave propagation in astrocytes. As the appearance and distribution of Annexin A7 during brain development has not been investigated so far, we focused on the distribution of Annexin A7 protein during mouse embryogenesis in the developing central nervous system and in the adult mouse brain.

**Results:**

Annexin A7 is expressed in cells of the developing brain where a change in its subcellular localization from cytoplasm to nucleus was observed. In the adult CNS, the subcellular distribution of Annexin A7 depends on the cell type. By immunohistochemistry analysis Annexin A7 was detected in the cytosol of undifferentiated cells at embryonic days E5–E8. At E11–E15 the protein is still present in the cytosol of cells predominantly located in the ventricular germinative zone surrounding the lateral ventricle. Later on, at embryonic day E16, Annexin A7 in cells of the intermediate and marginal zone of the neopallium translocates to the nucleus. Neuronal cells of all areas in the adult brain present Annexin A7 in the nucleus, whereas glial fibrillary acidic protein (GFAP)-positive astrocytes exhibit both, a cytoplasmic and nuclear staining. The presence of nuclear Annexin A7 was confirmed by extraction of the nucleoplasm from isolated nuclei obtained from neuronal and astroglial cell lines.

**Conclusion:**

We have demonstrated a translocation of Annexin A7 to nuclei of cells in early murine brain development and the presence of Annexin A7 in nuclei of neuronal cells in the adult animal. The role of Annexin A7 in nuclei of differentiating and mature neuronal cells remains elusive.

## Background

Annexins form a family of structurally related proteins, which bind to negatively charged phospholipids in a Ca^2+^-dependent manner [1]. They are characterized by a bipartite structure with a conserved C-terminal core domain and a unique N-terminal domain that varies in length and amino acid composition. The C-terminal domain is formed by either a four- or eightfold repeat of approximately 70 amino acids, each repeat carrying a Ca^2+^-binding site, and is responsible for phospholipid binding. The N-terminal regions are thought to confer functional diversity to the annexin proteins [2]. The biochemical features in vitro were analyzed extensively, but the in vivo functions of annexins remain unclear.

Annexin A7, the first annexin to be described, was isolated as the agent that mediated aggregation of chromaffin granules and fusion of membranes and phospholipids in the presence of Ca^2+^-ions [3]. Expression studies demonstrated the distribution of Annexin A7 in a wide variety of tissues and cells mainly enriched in the cytosol in close association with membranous structures, but it was also described in the nucleus of adrenal chromaffin cells [4]. The presence of an alternatively spliced cassette exon gives rise to two Annexin A7 isoforms corresponding in molecular mass to 47 kDa and 51 kDa. The isoforms differ in their N-terminal domain and exhibit a tissue-specific expression pattern. The 47 kDa isoform is present in all tissues except for skeletal muscle, where the 51 kDa isoform is exclusively present. Heart muscle, brain tissue and red blood cells contain both isoforms [5-8]. Previous studies indicated that the subcellular localization of Annexin A7 changes during myoblast differentiation. In undifferentiated cells the protein is equally distributed between cytosol and membrane fractions while in differentiated cells it is exclusively present in the membrane fraction [7]. Reports by Clemen et al. [9] and Herr et al. [8,10] demonstrated roles for Annexin A7 in shape and osmotic resistance of red blood cells, platelet aggregation velocity, and in the velocity of spreading astrocytic Ca^2+^-waves. Annexin A7 is also involved in the maintenance of regular cardiac electrophysiology and Ca^2+^-homeostasis [Schrickel et al., submitted]. Detailed reports on appearance and distribution of Annexin A7 during brain development are not available. In the present study we focus on the distribution of Annexin A7 in the developing brain of mice embryos between E5 and E16, and in the adult mouse brain.

## Results

### Annexin A7 is expressed in the early mouse embryo

**Figure 1:**
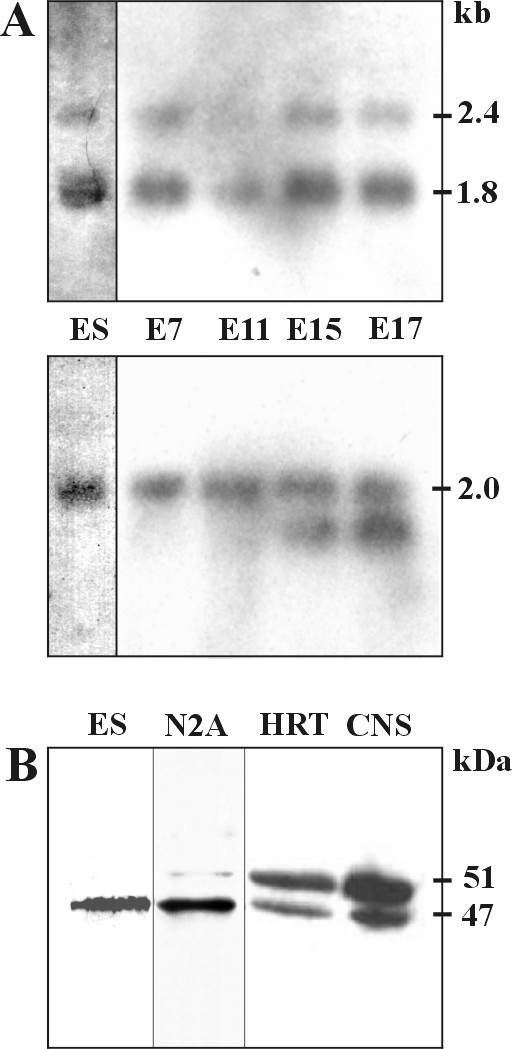
**Expression of Annexin A7. **(**A**) Northern blot analysis of RNA from ES-cells and embryos E7, E11, E15, E17 probed with a full length annexin A7 cDNA. Transcripts of 1.8 and 2.4 kb are detected in all stages examined (upper panel). Reprobing with a β-actin probe verified equal loading (lower panel, 2.0 kb band). (**B**) Murine ES cells (ES) express only the small Annexin A7 isoform. Both isoforms are expressed in neuroblastoma neuro-2a cells (N2A), heart (HRT) and brain tissue (CNS) from adult mice. Proteins from neuro-2a and ES cells, heart and brain were resolved by SDS polyacrylamide gel electrophoresis (12% acrylamide) and transferred to nitrocellulose membranes. The Western blot was probed with mAb 203–217 followed by incubation with a peroxidase coupled secondary antibody. Detection was with enhanced chemiluminescence.

First we examined the expression of Annexin A7 in ES cells (Bruce4, established from C57BL/6J mice) and the early stages of mouse embryonic development at the mRNA level by northern blot analysis and at the protein level by Western blotting and immunohistochemistry, respectively. Northern blot analysis shows in ES-cells and at embryonic days E7, E11, E15, and E17 two mRNA species of 1.8 kb and 2.4 kb, which result from alternative splicing in the untranslated 3'end (Fig. 1A) [11]. We found similar Annexin A7 mRNA levels in the four embryonic stages. Reprobing with a β-actin probe verified equal loading; the appearance of a faster migrating mRNA species in addition to the 2.0 kb species is characteristic for β-actin [12]. On the protein level, ES cells express only the smaller Annexin A7 isoform of 47 kDa (Fig. 1B, neuro-2a cells and heart tissue are included for control).

**Figure 2:**
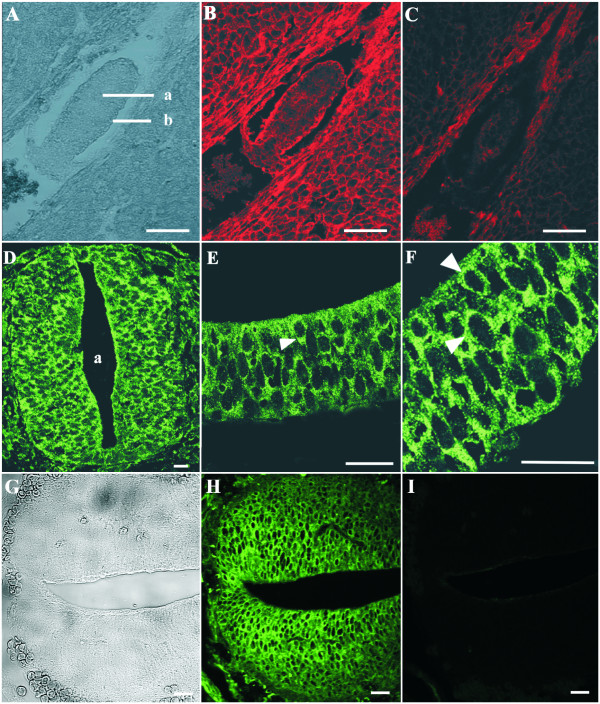
**Annexin A7 immunoreactivity in early mouse embryos. **(**A**) Phase contrast, embryo E5: The egg cylinder consists of an inner cell mass (a) representing the ectoderm and an outer layer of endoderm cells (b). **(B) **Immunostaining of the paraffin section was performed using purified mAb 203–217 and Cy3-conjugated anti-mouse IgG. Annexin A7 is expressed in both cell types of the egg cylinder with a strong staining of the endoderm and a weaker staining of the ectoderm. The nuclei are devoid of immune reactions. (**C**) Negative control using the secondary Cy3-antibody only. (**D-F**) Annexin A7 expression in the proximal neural tube (D) and nearby neural fold (E,F), embryo E8, transverse section. Immunolabeling of Annexin A7 was performed with purified mAb 203–217 and visualization was with an Alexa Fluor 488-conjugated anti-mouse IgG. (**D**) An intense Annexin A7 immunostaining is detectable in the neuroepithelium of the neural tube (a, lumen of neural tube). (**E,F**) Higher magnifications of the neuroepithelium show that Annexin A7 is expressed in the cytosol. Arrowheads point to Annexin A7 immunoreactivity in the cytosol. (**G**) Phase contrast, embryo E13, caudal neural tube. **(H) **Immunostaining of the paraffin section was performed using purified mAb 203–217 and Alexa Fluor 488-conjugated anti-mouse IgG. (**I**) Negative control using the secondary Alexa Fluor 488-antibody only. Bar, 20 μm.

Immunohistochemistry confirmed the presence of the protein during early development. Annexin A7 immunoreactivity using mAb 203–217, which specifically recognizes both isoforms of Annexin A7, can be observed in the two cell types of the cylinder stage at E5 with a weaker Annexin A7 expression in the ectoderm (Fig. 2A–C). At E8 Annexin A7 can be detected in almost all tissues of the embryo (data not shown). A correlation between the presence of Annexin A7 and the origin of a cell type from one of the three germ layers is not apparent. We specifically noted the localization of the protein in the neuroepithelium of the neural fold and tube (Fig. 2D–F, proximal, E8; Fig. 2G–I, distal, E13). At the subcellular level, Annexin A7 was present in punctate structures mainly in the cytosol of all examined cells (Fig. 2E,F). This distribution was also observed during subsequent development.

### Annexin A7 changes its subcellular localization in the embryonic brain at E16

**Figure 3:**
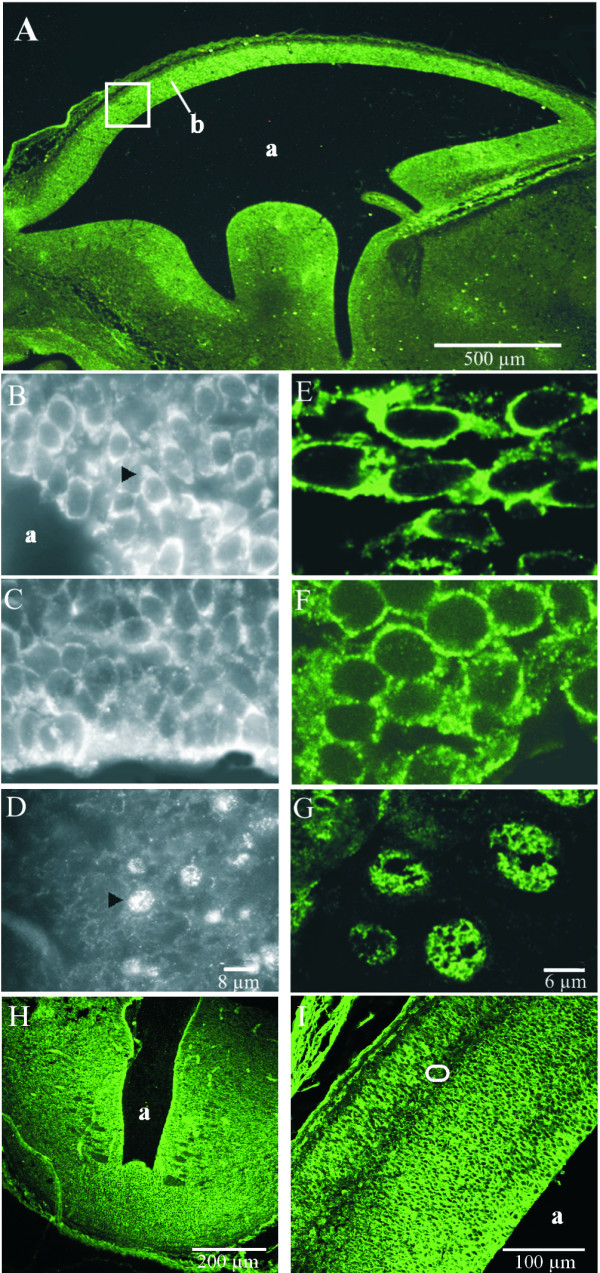
**Subcellular localization of Annexin A7 in embryos E13, E15 and E16. **Paraffin sections of embryonic brain were stained with purified mAb 203–217. Annexin A7 was visualized with Alexa Fluor 488-conjugated secondary antibody. (**A**) Overview, at E16 the immature GFAP-negative cells of the forming cerebral neocortex (b) surrounding the lateral ventricle (a) are strongly stained; square, a higher magnification of this area is given in (I). (**B**) Higher magnification of the earlier stage E13 (H) shows, that the cells are stained in the cytosol (arrowhead). (**C**) Two days later at E15 the cells have rounded up, and Annexin A7 stays in the cytosol. (**D**) At E16 the first nuclear staining becomes apparent (arrowhead) in cells of the intermediate zone located between ventricular germinative zone and marginal neopallial cortex as seen in (I), oval. (**E-G**) Confocal microscopy confirms the results in B-D. (**H**) Overview, at E13 the immature GFAP-negative cells are strongly stained; a, lateral ventricle. (**I**) Higher magnification of a cortical section of (A, square) demonstrating ventricular, intermediate, and marginal zones; oval, a higher magnification of this area is given in (D,G).

Next we analysed the cellular and subcellular distribution of Annexin A7 in the developing mouse brain (Fig. 3). At E13–E16 in transverse sections of the embryonic brain, most of the immunoreactive cells are present in the ventricular germinative zone surrounding the lateral ventricle (Fig. 3A, overview E16; Fig. 3H, overview E13). A closer inspection revealed that Annexin A7 is mainly localized in the cytosol at E13 (Fig. 3B,E). During further development the immature cells have rounded up two days later, but they retain Annexin A7 in the cytosol at E15 (Fig. 3C,F). At E16 we observed the first prominent nuclear staining of Annexin A7 (Fig. 3D,G) in cells of the intermediate zone (Fig. 3I, oval), which contains neurons radially migrating towards the growing neopallium cortex [13,14] and also glial cells forming the white matter of the adult cortex. Cells located marginally in the neopallial cortex also exhibit a nuclear stain. Labelling in the ventricular germinative zone is less prominent and the cytosol is no longer more strongly stained than the nucleus.

### Mature neurons in adult mouse brain show an intense nuclear staining of Annexin A7

**Figure 4:**
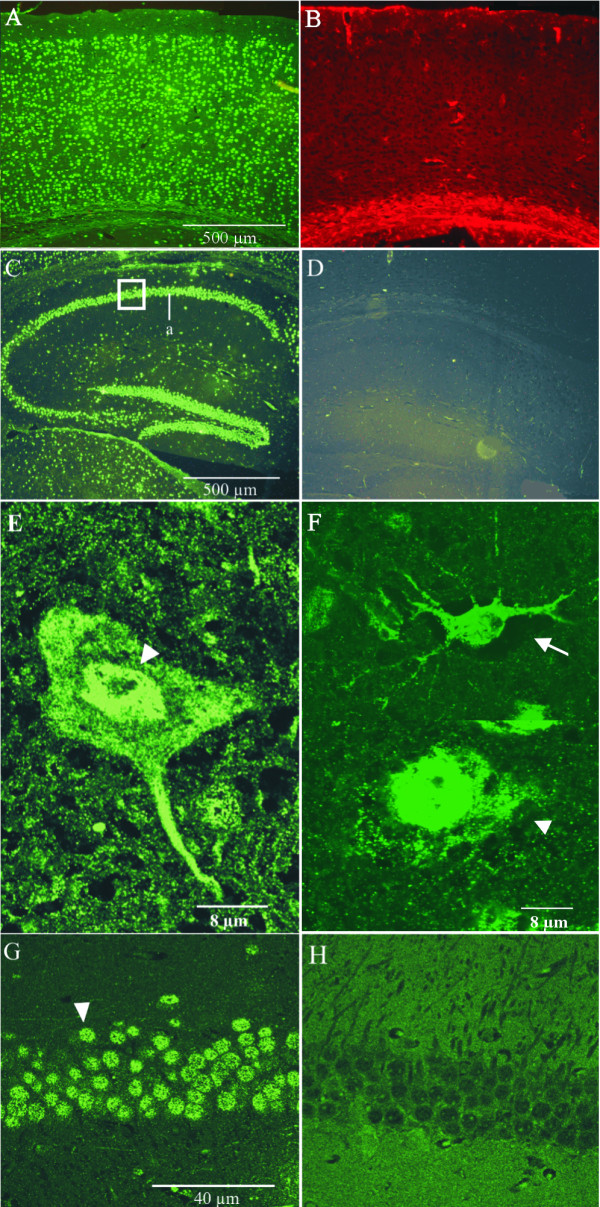
**Annexin A7 is present in neurons and astrocytes of the cortex temporalis and hippocampal formation of 10-weeks-old mice. **(**A**) Low magnification of the cortex temporalis presents an Annexin A7 expression in cells of the pial border, in neurons of all six isocortical laminae, and a weak signal in the adjacent white matter. (**B**) Corresponding section stained with GFAP. (**C**) Staining in the Stratum pyramidalis (a) and in the dentate gyrus of the hippocampus; square, a higher magnification of acorresponding area is given in (G,H). An intense Annexin A7 immunostaining is detectable. (**D**) Corresponding section stained with secondary antibody only. (**E**) Presence of Annexin A7 in pyramidal neurons (lamina pyramidalis externa) of the isocortex temporalis. These neurons were identified based on their morphology, distribution and lack of GFAP staining. AnnexinA7 exhibits a punctate staining, which is pronounced in the nucleus (arrowhead). (**F**) Higher magnification of image (A) also shows Annexin A7 in nuclei of neurons (lamina granularis externa (corpuscularis), arrowhead) and in the cytoplasm and nuclei of astrocytes (lamina molecularis, arrow; GFAP-confirmed). (**G**) Higher magnification of the pyramidal neurons in the hippocampus confirms the presence of the Annexin A7 protein in the nucleus (arrowhead) of mature neurons. (**H**) To further confirm this, a similar section derived from an *AnxA7*^-/- ^mouse was stained with the annexin specific antibody and lacked the nuclear signal. The residual stain of the tissue is unspecific, as it is also observed in controls of the *AnxA7*^-/- ^brain omitting the primary antibody (data not shown). All paraffin sections were stained with mAb 203–217 (A, C, E, F, G) or anti-GFAP-antibody (B). The hippocampal control section (D) lacks the primary antibody.

Both Annexin A7 isoforms are found in adult brain tissue (Fig. 1B). In general, we observed two characteristic staining patterns for Annexin A7, a prominent nuclear stain in neurons (determined by lack of GFAP-immunoreactivity, localization, morphology) and a cytoplasmic and nuclear stain in astrocytes (GFAP-positive) in all areas of the mature murine brain. In the neocortex (isocortex) Annexin A7 was strongly enriched in nuclei of neurons of all six cortical laminae (Fig. 4A, section derived from cortex temporalis). For control, in the adult brain type-1 astrocytes can be characterized by a positive GFAP-stain (Fig. 4B). Fig. 4F demonstrates the presence of nuclear Annexin A7 in a neocortical neuron of the external granular layer (layer II) and in an astrocytic cell of the neocortical molecular layer (layer I) which additionally exhibits an intense signal in the cytoplasm and in cellular branches. A further cell type of the isocortex showed a strong immunoreactivity in both, nucleus and cytosol, and was identified as pyramidal neuron based on morphology and absence of GFAP immunoreactivity. However, Annexin A7 apparently is more enriched in the nucleus (Fig. 4E).

In the hippocampal formation we detected prominent Annexin A7 immunostaining in the stratum pyramidalis and in the dentate gyrus and also a weak astrocytic staining (Fig. 4C). The astrocytes are mainly localized in the area between cells exhibiting nuclear Annexin A7 staining and show the protein also in the nucleus and the cytoplasm (data not shown). When we analyzed the pyramidal neurons in the hippocampus at a higher magnification we found that Annexin A7 again mainly localized to the nuclei (Fig. 4G). Polyclonal antibodies gave a similar staining pattern (data not shown). The intense nuclear staining was absent in controls either stained only with the secondary antibody (Fig. 4D) or in brain sections derived from *AnxA7*^-/- ^mice stained for Annexin A7 (Fig. 4H). The residual staining seen in the *AnxA7*^-/- ^brain is unspecific as it is also present in corresponding negative controls lacking the primary antibody. The absence of Annexin A7 protein in brain and other tissues of the *AnxA7*^-/- ^mouse has been verified in [10]. In another actual study of the knock out mouse we detected a thickened basal membrane and a widened intercellular space. This may cause unspecific binding of the secondary antibody.

**Figure 5:**
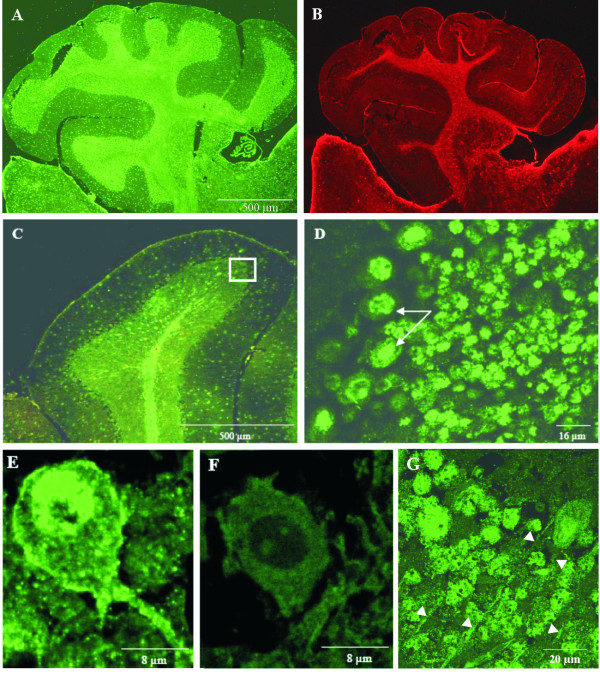
**Annexin A7 immunostaining in the cerebellum of adult mice. **(**A**) Low magnification of the cerebellum presents an Annexin A7 expression mainly in cells of the stratum granulosum and laminae medullares. (**B**) Corresponding section stained for GFAP. (**C**) Higher magnification of folia of the cerebellum, where a polyclonal anti-AnnexinA7 antibody was used; square, a higher magnification of acorresponding area stained with mAb 203–217 is given in (D). Between stratum granulosum and stratum moleculare the layer of Purkinje-cells (stratum neuronorum piriformium (ganglionare)) can be observed. (**D**) Staining of the band of Purkinje-cells (arrows). The positive Annexin A7-stain in the stratum granulosum is due to staining of the nuclei of neurons. (**E**) In addition to an intense staining of the nucleus, the cell body is AnnexinA7-positive including both dendrites of the Purkinje-cell shown. (**F**) Corresponding section from an *AnxA7*^-/- ^mouse. (**G**) AnnexinA7 staining of axons (arrowheads) running from the laminae medullares to the Purkinje-cell layer located in the round end of a convolution. Sections A, D, E, F, G were stained with mAb 203–217.

An equal staining of Annexin A7 was also found in the cerebellum (Fig. 5A). Nuclei of neurons in the stratum granulosum show the most prominent staining (Fig. 5C,D). In the stratum moleculare, which is poor in cell bodies of neurons, only a few dots appear which also correspond to nuclei of neurons. Astrocytes are also positive for Annexin A7 in the nuclei and cytoplasm (data not shown). The signal of the pial border and the prominent stain of the white matter tracts (laminae medullares) are due to Annexin A7 positive astrocytes (Fig. 5 A–C). Higher magnification of the boundary layer between stratum moleculare and granulosum (Fig. 5D) revealed an Annexin A7 signal in nuclei, but also in the cytoplasm and at the plasma membrane of Purkinje cells (Fig. 5E). The typical pair of dendrites points to the margin of the cerebellar cortex. The specificity of the Annexin A7 signal was further confirmed in similar brain sections of the *AnxA7*^-/- ^mouse (Fig. 5F). Fig. 5G shows Annexin A7 in neurites (axons) connecting the laminae medullares with the Purkinje-cell layer. Most likely these are the axons of the Purkinje-cells. Apart from these efferent neurites the laminae medullares (lamina alba, white matter) contain afferent mossy and climbing fibers. Thus, the intense stain of the cerebellar white matter arises from Annexin A7 located in astrocytes and neurites.

### Expression of Annexin A7 in the adult human isocortex

**Figure 6:**
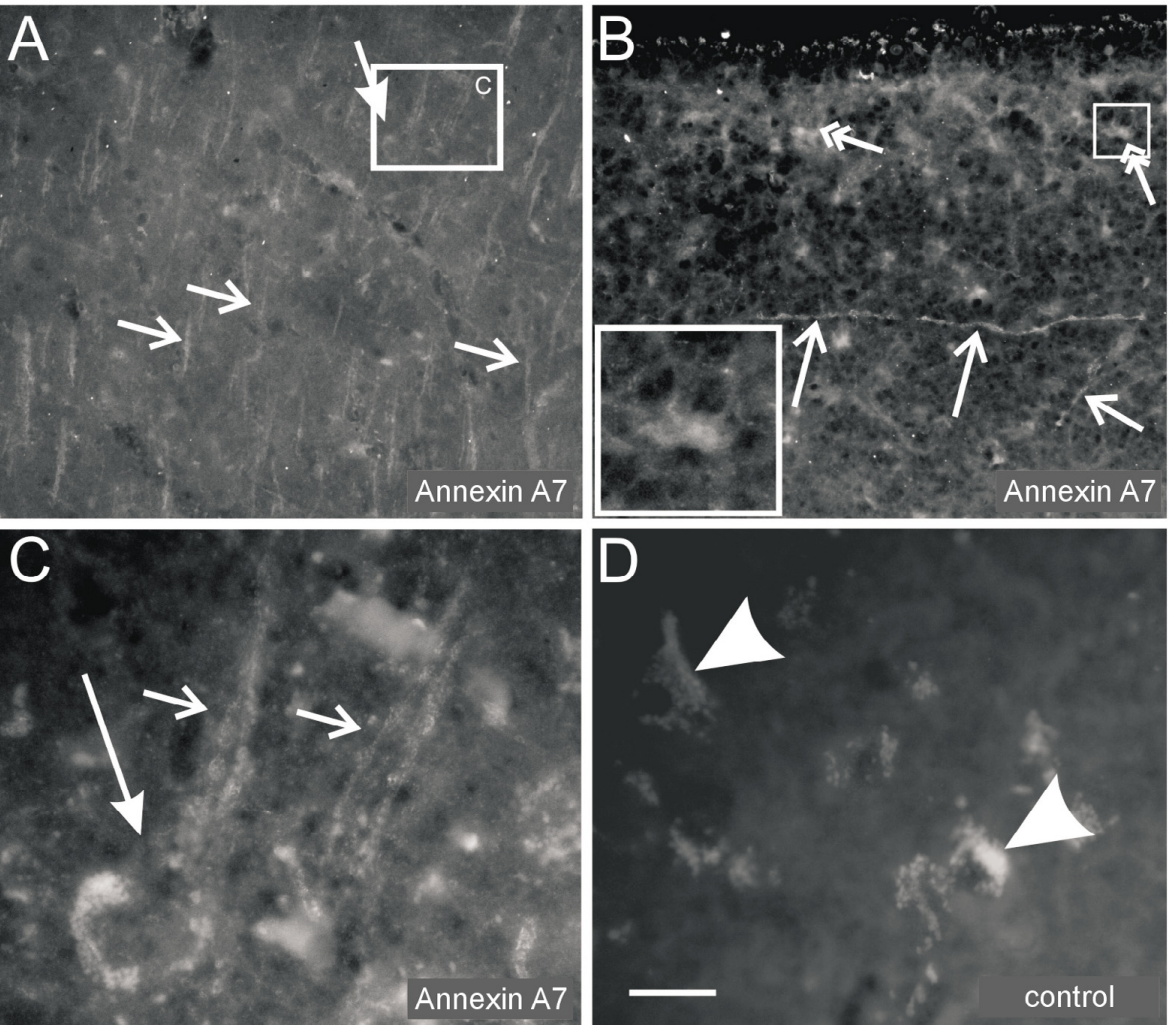
**Annexin A7 immunostaining in human isocortex. **(**A**) Pyramidal neurons (bold arrow) and apical dendrites (small arrows) were clearly labeled with the antibody against Annexin A7 in the human parietal cortex. (**B**) A dendritic staining was also seen in the molecular layer (small arrows). In addition the subpial astrocytes were weakly labeled (double headed arrows). The staining was cytoplasmic, only few astrocytes showed a nuclear staining as well (inset). (**C**) Enlargement of (A): Annexin A7 was seen at the cell membrane of the perikaryon (bold arrow) and the apical dendrite (small arrows). (**D**) A blank control lacking the primary antibody did not show a specific labeling, but autofluorescent lipofuscin was detectable in the neurons (arrowheads). Bar, (**A) **70 μm, (**B) **50 μm, (**B**-inset, **C) **17 μm, (**D) **20 μm.

In the human parietal neocortex of aged individuals without any neuropathological alterations, subpial astrocytes exhibited a staining of Annexin A7 in the cytoplasm. A nuclear presence of Annexin A7 was limited to single astrocytes (Fig. 6B). Pyramidal neurons, predominantly those of layer V exhibited Annexin A7 at the plasma membrane of their perikaryon as well as of the apical dendrite (Fig. 6A,C). The neurons lacked a signal for Annexin A7 in their nuclei. Apical dendrites within the molecular layer also indicated a positive staining for Annexin A7 (Fig. 6B). The staining pattern of Annexin A7 in the human autopsy brain did not change after pre-treatment with trypsin.

### Presence of Annexin A7 in nuclei from neuronal and astroglial cells

The nuclear localisation of Annexin A7 in mice observed in immature cells at E16 and differentiated adult neurons could be only detected as a strong signal when the brain sections were pre-treated with trypsin before antibody staining. This procedure is thought to allow the antibodies to access epitopes masked by the formaldehyde fixation. Methanol fixation of cultured cells led to inconsistent results. Antigen retrieval in formalin-fixed and paraffin-embedded tissue sections employs various heating or proteolytic pre-treatment methods [[15-17]; Ein Handbuch für die Histologie, dianova GmbH, Hamburg, Germany]. These methods can result in moderate or strong specific antibody staining, but the detectability of other antigens might be decreased. Moreover, the optimal pre-treatment has to be individualised for each antigen.

**Figure 7:**
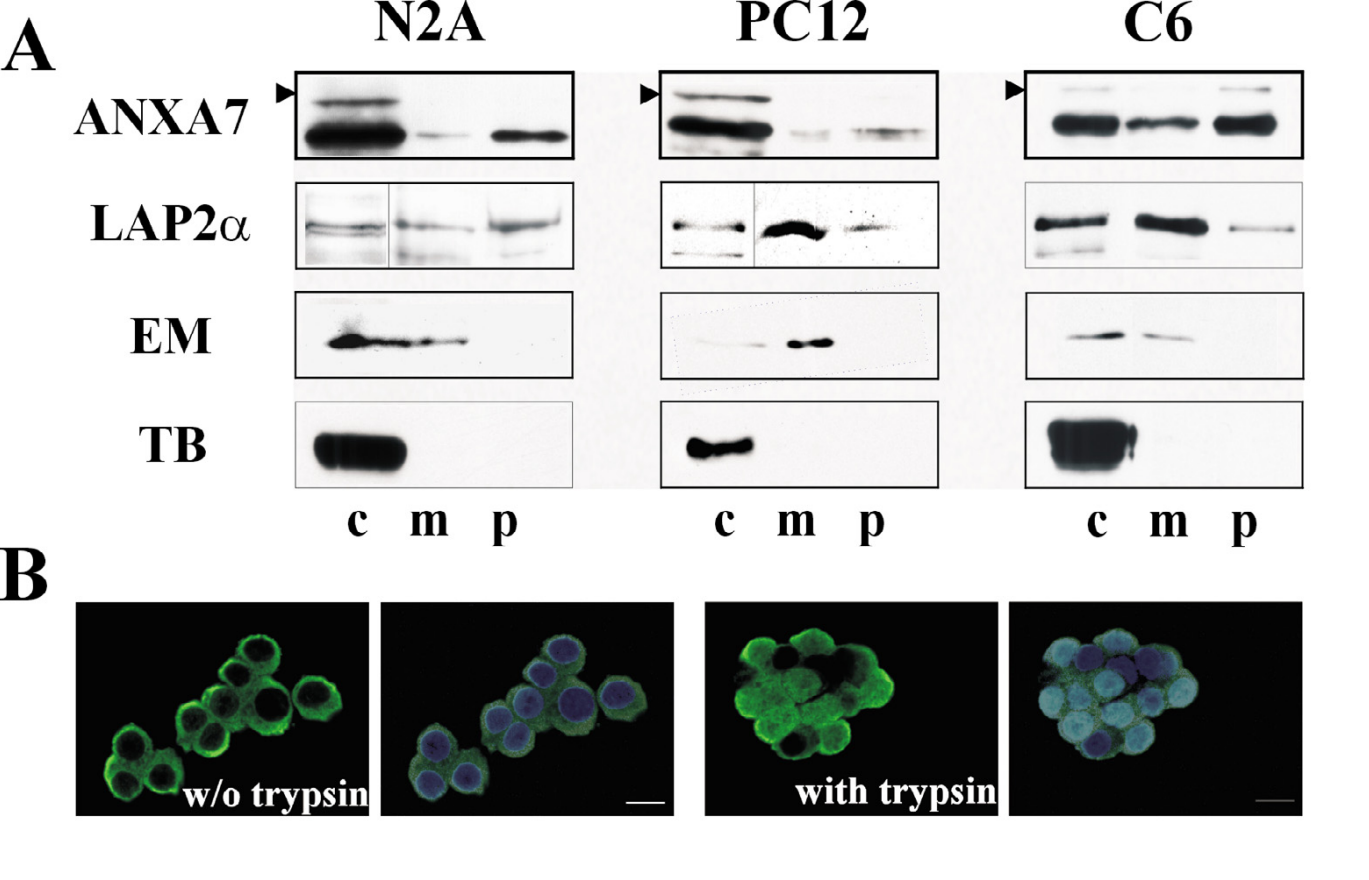
**Nuclear localization of Annexin A7. (A) **Extraction of Annexin A7 from nuclei of neuronal (PC-12, N2A) and an astroglial (C6) cell line using a hypotonic buffer. Samples of total cells (c), and corresponding amounts of nuclear membranes (m) and nucleoplasm (p) were subjected to SDS-PAGE and Western blotting. Annexin A7 (AnxA7) isoforms are extractable from the nucleus of the indicated cells (47 kDa and 51 kDa isoform, arrowhead). For control, immunoblotting of LAP2α (75 kDa; non-membrane-bound isoform), Emerin (EM, 34 kDa), and tubulin (TB, 55 kDa) which are specific for nucleoplasm (LAP2α), nuclear membranes (Emerin), and the cytoplasm (tubulin) are shown. Note that the nucleoplasm is only partially extractable. **(B) **Immunofluorescence images of the PC-12 cells used. Cells were fixed with paraformaldehyde, permeabilized with Triton X-100, pretreated with trypsin as indicated, and stained with Annexin A7 specific mAb 203–217, DAPI in blue, bar 10 μm.

To verify the presence of endogenous Annexin A7 in nuclei, we used a biochemical approach and purified nuclei from neuro-2a, PC-12, and C6 cells and treated them with a hypotonic extraction buffer to obtain the nucleoplasm (Fig. 7A). The nucleoplasm and the remaining nuclei, from which the nucleoplasm has been extracted partially, were subjected to SDS-PAGE and Western blotting. Western blots probed with mAb 203–217 showed Annexin A7 in the nucleoplasm of neuronal (neuro-2a, PC-12) and astroglial (C6) cell lines (Fig. 7A), however, the large isoform could only be clearly extracted with nucleoplasm from C6 cells. For control, antibodies against LAP2α, Emerin and tubulin were used to verify that the nucleoplasm (marker:LAP2α, which is anon-membrane-bound isoform of LAP2) was successfully extracted and also not contaminated by nuclear membranes (marker:Emerin) or by cytoplasm (marker:tubulin). Likewise, in these neuronal and astroglial cell lines Annexin A7 had been observed in the nucleus by immunofluorescence (Fig. 7B and data not shown). We noticed no difference between PC-12 and neuro-2a cells, however, as for the brain sections, we found an increase of the Annexin A7 staining after pre-treatment with trypsin.

## Discussion

In the present study we explored the appearance of Annexin A7 during mouse development at the mRNA and protein level and focused on the central nervous system during embryogenesis. Northern blot and immunohistochemistry analysis show the expression of Annexin A7 in all embryonic tissues from day E5 on, the earliest day studied. Differentiating cells, like the neural ectoderm, which is the origin of the cells belonging to the nervous system, show Annexin A7 immunoreactivity mainly in the cytosol. Endodermal as well as mesodermal cells exhibit a similar subcellular localization of the protein.

In the developing brain we noted a striking change in the subcellular distribution of Annexin A7. Cells in the stratum germinativum of the neopallial cortex, which surrounds the lateral ventricle, show at E13 and E15 a staining for Annexin A7 mainly in the cytosol. But this staining largely disappears and at the following day E16 we detect Annexin A7 in the nucleus of cells in the intermediate and marginal zone of the neopallium. The different expression patterns of Annexin A7-positive cells from the ventricular germinative zone to the marginal zone of the later neocortex observed in this study are similar to the developmental patterns of Tenascin-C-positive astroglial precursors following the guidance of the radial glial cells [18]. Moreover, the patterns resemble that of migrating and differentiated neurons described by Berry et al. [13]. Neurons that are generated prenatally in the proliferative ventricular layer of the neopallial cortex subsequently migrate through the intermediate zone to form the different cortical cell layers in a declining inside-out gradient of cell maturation. These observations suggested that the subcellular localization of Annexin A7 depends on developmental stage and cell type.

In the adult brain we generally observed a nuclear localization for Annexin A7 in neurons whereas astrocytes exhibited both a cytosolic as well as a nuclear staining. However, pyramidal neurons of the isocortex and Purkinje-cells of the cerebellum exhibited a cytosolic stain of intermediate intensity including their neurites and dendrites. We have previously reported the expression of Annexin A7 in human temporal neocortex and hippocampus obtained from neurosurgery for therapy-refractory epilepsy and found the two Annexin A7 isoforms restricted to the cytoplasm and nuclei of astrocytes, whereas neurons lacked any signal [19]. The hippocampal area showed typical signs of Ammon's horn sclerosis, but the adjacent temporal neocortex tissue did not show any histopathological alterations. This data is in discrepancy with our actual observation in the murine brain. To determine if this is due to different methods in immunofluorescence staining or indeed different expression patterns in mouse and human, we repeated the immunofluorescence of human brain. This time we investigated sections from human parietal cortex of autopsy brain. The astroglial expression of Annexin A7 could be confirmed, although not all astrocytes exhibited the nuclear staining. Pyramidal neurons however indicated a distinct staining of Annexin A7 most prominent along their dendrites. The different results obtained for the neurons may be explained by the fact that we previously used frozen tissue sections or that they were derived from patients suffering from temporal lobe epilepsia. On the other hand the actual human brain tissue used was from aged patients and did not correspond to the age of the mice included in this study. In cultured cells after cell damage or apoptosis (unpublished observations) or in cells treated with a Ca^2+^-ionophore Annexin A7 translocated from the cytoplasm to cellular membranes [19]. We therefore favor the hypothesis that Annexin A7 in the sensitive neurons of the human autopsy brain may have similarly translocated to the cellular membrane. This property of translocation and membrane binding is common to all annexins and commercially available kits for apoptosis detection employ recombinant AnnexinA5. The presence of nuclear Annexin A7 in murine brain was confirmed by controls using sections from the *AnxA7*^-/- ^mouse and by a biochemical extraction of the protein from the nucleus. Both Annexin A7 isoforms described in brain tissue seemingly are expressed by neurons and astrocytes, which was shown using total cell extracts of cultured neuo-2a, PC-12, and C6 cells. In addition to their expression in brain, both Annexin A7 isoforms have only been described in heart muscle and red blood cells [5-8].

Although the inactivation of the annexin A7 gene did not interfere with the viability and development of knock out mice [10], their generation allowed us to address the role of Annexin A7 in specific cell types [8,9]. Indeed, when we analyzed primary astrocytes from an *AnxA7*^-/- ^mouse for Ca^2+^-dependent signaling processes, we found that they exhibited a significantly increased velocity of mechanically induced astrocytic Ca^2+^-waves as compared to wild type [9]. This led us to propose, that Annexin A7 can act as a Ca^2+^-buffer and is involved in Ca^2+^-homeostasis. In neurons Ca^2+ ^ions play major roles in various physiological and pathophysiological processes [20-25]. One can speculate about an involvement of Annexin A7 in the regulation of these Ca^2+^-dependent processes, propositions that need further investigation. However, such roles are confirmed for heart function by studies of Schrickel et al. [submitted], who described an involvement of Annexin A7 in the maintenance of a regular cardiac electrophysiology and Ca^2+^-homeostasis.

An altered subcellular location during embryogenesis was also reported for Annexin A11. The developing gray matter of the rat embryonic spinal cord exhibited primarily nuclear localization of Annexin A11, while immunoreactivity was lost from the nuclei in the adult spinal cord [26]. In contrast, our studies show a relocation of Annexin A7 from the cytosol to the nucleus in cells of the embryonic neuronal tissue. The Annexin A7 distribution is determined by a variety of factors. An important one appears to be Ca^2+^, which promotes binding of Annexin A7 to membranes and also allows aggregation of annexins [27]. Binding partners of Annexin A7 such as sorcin might represent additional factors [28]. Although the members of the annexin family are generally found at the plasma membrane, in the cytoplasm or in association with the cytoskeleton, Annexins A1, A2, A4, A5 and A11 have been described to be localized at least partially in the nucleus [26,29,30]. Studies with human foreskin fibroblasts demonstrated that Annexin A1, A4 and A5 are all present in the nucleus at higher concentration than in the cytosol [31]. Raising intracellular Ca^2+ ^led to relocation of these annexins to the nuclear membrane. An important role for annexins in mediating the Ca^2+^-signal within the nuclei of the fibroblasts was proposed. These results mirror studies with stably transfected C6 cells, in which high intracellular Ca^2+^-concentrations induced a marked redistribution of Annexin A7 from its localization in the nucleoplasm to the nuclear membrane [19].

None of the annexins contains a typical nuclear localization signal and their mechanism of nuclear import remains to be elucidated. For Annexin A11 it was shown that nuclear localization is mediated by its N-terminal region, which also contains a binding side for the S100 protein calcyclin [29]. More recently Tomas and Moss [32] showed, that Annexin A11 and S100A6 assemble at the nuclear envelope during nuclear breakdown. Their role in this process is not known. In general it seems that the nuclear localization of the annexins is actively regulated. For example the nucleocytoplasmic compartmentalization of Annexin A2 is controlled by sequestration of the AnxA2/p11 complex modulated by phosphorylation and by a nuclear export signal found in the AnxA2 3–12 region [33]. One function of Annexin A2 in the nucleus, that appears not to involve binding of p11, has been suggested by its purification as part of a primer recognition protein complex that enhances DNA polymeraseα-activity in vitro [34]. The annexins may participate in a nuclear response to initial cell stimulation or to a Ca^2+^-transient, presumably by regulating DNA replication. For Annexin A7 such a pathway is very speculative at the moment. Future studies are however directed by these findings and will concentrate on the identification of the nuclear localization signal of Annexin A7 as well as on the role of Annexin A7 in the nuclear compartment.

## Conclusion

In this article we report the translocation of Annexin A7 to nuclei of differentiating cells in the developing murine brain. In the adult brain Annexin A7 generally was detected in nuclei of neurons. Astrocytes, cerebellar Purkinje-cells and neocortical pyramidal neurons exhibited both, Annexin A7 in the cytosol and in the nuclei. Thus, the subcellular localisation of Annexin A7 depends on the developmental stage and the cell type. A role of Annexin A7 as well as of other annexins in nuclei remains speculative. The results obtained by immunofluorescence were confirmed by extraction of Annexin A7 from nuclei of neuronal and glial cell lines.

## Methods

### Animals and tissue preparation

129SV strain mice were used. The day on which the vaginal plug was confirmed was defined as E0. All the animal experiments were performed in accordance with the Guide for the Care and Use of Laboratory Animals (NIH Publication No. 85-23). Pregnant females were sacrificed by cervical dislocation at various intervals between E5 and E17. Before E9, embryos were embedded within the maternal uterus. After E9, embryos were removed from their membranes and embedded separately. Experiments were performed with four to six animals at each developmental stage. The mature brain used was derived from mice of three to four month of age. The embryos and tissues were fixed in freshly prepared 4% paraformaldehyde solution for 2–12 h depending on the tissue thickness [35]. They were dehydrated with ethanol and embedded in paraffin according to standard procedures. Microtome sections were cut at 7–10 μm, placed on slides and stored until further processing.

### Human brain tissue

Five human autopsy brains of male and female patients without any neuropathological changes (age 61–77 years; post mortem interval: 12–72 h) were fixed in an aqueous solution of formaldehyde for three weeks. Blocks of the parietal neocortex were embedded in paraffin and microtomed at 12 μm thickness. Sections were stained with aldehydefuchsin Darrow-red to confirm the absence of neuropathological alterations. All procedures were conducted in accordance with the Declaration of Helsinki and approved by the ethics committee of the University of Bonn Medical Center.

### Antibodies

Antibodies used in this study were mAb 203–217 directed against the core domain of mouse Annexin A7 [6] (purified IgG, 1:50) specifically recognizing both Annexin A7 isoforms [[6,10], and data not shown], a polyclonal antibody against mouse Annexin A7 [10] (1:200), a polyclonal antiserum raised against the astrocyte-specific intermediate filament glial fibrillary acidic protein (GFAP; DAKO, 1:400), anti-β-tubulin (Sigma, 1:1000), anti-Emerin (novocastra, 1:2000), and polyclonal anti-LAP2α (kindly provided by Ronald Foisner, 1:5000).

### Immunofluorescence microscopy

Immunohistochemical staining using mAb 203–217 as the primary antibody was with AlexaFluor488-conjugated goat anti-mouse IgG (Molecular Probes, Leiden, The Nether-lands) or Cy3-labeled goat anti-mouse IgG as the secondary antibodies. Polyclonal anti-Annexin A7 antibody and anti-GFAP-antibody were combined with Alexa Fluor 568-conjugated goat anti-rabbit IgG (Molecular Probes). The specific labelling of Annexin A7 detected with the anti-mouse-IgG secondary antibodies in mouse tissue sections was confirmed using primary antibodies, which were directly coupled to Alexa Fluor488.

The paraffin-embedded sections were first treated three times for 2 min in xylene to remove paraffin and then rehydrated step by step with descending concentrations of ethanol for antibody staining [35]. After three washings in PBS (137 mM NaCl, 2.7 mM KCl, 8.1 mM Na_2_HPO_4_, 1.5 mM KH_2_PO_4_, pH 7.4), the tissue sections were incubated in PBS containing 0.1% trypsin for 10 min at room temperature to free formaldehyde-crosslinked epitopes [15-17]. After additional three washes with PBS, non-specific binding sites were blocked by incubating the slides with PBG (0.5 % BSA, 0.045 % fish gelatine in PBS) in 10% normal goat serum (NGS) for 2 h. Incubation with the primary antibodies was performed at 4°C overnight diluted in PBG supplemented with 1 % NGS. After five washes in PBG for 5 min each, the slides were incubated for 1 h with the secondary antibody at room temperature, washed three times in PBG and three times in PBS, 5 min each, rinsed in water and embedded in gelvatol [36]. For control, sections were incubated only with the secondary antibody or sections obtained from an annexin A7 knockout mouse were used [10]. Samples were examined with a confocal laser scan microscope (Leica TCS SP; Leica, Bensheim, FRG).

### Immunofluorescence microscopy of human brain

The paraffin-embedded human brain sections were deparaffinzed likewise. Immunostaining for Annexin A7 was performed using the monoclonal antibody mAb 203–217. Microwave pre-treatment was used for all sections [15]. Trypsin pre-treatment was used as indicated. Carbocyanine 2 and 3 labeled anti-mouse IgG secondary antibodies (Dianova, Hamburg, Germany) were used to detect the primary antibody and showed similar staining patterns. Blank controls were performed, too.

### Cell culture and growth conditions

The cell lines used were cultured according to the instructions provided by ATCC: murine neuroblastoma neuro-2a (ATCC CCL-131), rat pheochromocytoma PC-12 (ATCC CRL-1721), and rat glioblastoma C6 (ATCC CCL-107). One day before fixation with 4%paraformaldehyde and permeabilization with 0.5% Triton X-100, cells were seeded on coverslips. Immunostaining was performed as described for the tissue sections with or without the trypsin treatment.

### Extraction of nuclear Annexin A7

Cultured cells were trypsinized, counted and washed once in PBS. They were resuspended in buffer1 (0.32 M sucrose, 10 mM Tris, pH8.0, 3 mM CaCl_2_, 2 mM Mg-acetate, 0.1 mM EDTA, 0.5% NP40, 1 mM DTT, 0.5 mM PMSF; 1 × 10^7^cells/400μl buffer) and sonified on ice (sonifier UP200S, dr.hielscher, 40 s, amplitude 50%, cycle 0.5). This step was repeated until no more intact cells were observed by light microscopy. The resulting homogenate was centrifuged at 500 g for 5 minutes, washed twice in 1 ml buffer 1 without NP40 and centrifuged again. The resulting pellet contains purified nuclei. For partial extraction of the nucleoplasm according to [37], nuclei were resuspended in buffer 2 (20 mM Hepes, pH 7.9, 1.5 mM MgCl_2_, 20 mM KCl, 0.2 mM EDTA, 25%v/v glycerine, 0.5 mM DTT, 0.5 mM PMSF; nuclei of 10^7^cells/30 μl buffer) and incubated on ice for 30 minutes. An equal volume of buffer 3 was added in aliquots (20 mM Hepes, pH 7.9, 1.5 mM MgCl_2_, 800 mM KCl, 0.2 mM EDTA, 25%v/v glycerine, 0.5 mM DTT, 0.5 mM PMSF, 1% NP40, protease inhibitor cocktail (Sigma)) and the solution was incubated while shaking for further 30 minutes on ice. Then the solution was centrifuged for 15 minutes at 14.000 g, the resulting supernatant was used as nucleoplasm, and the pellet was washed once and adjusted to an equal volume with PBS. Nucleoplasm and nuclear pellet were subjected to SDS-PAGE and Western blotting.

### Western blotting

Cells or cellular fractions were lysed in SDS sample buffer and homogenates were subjected to SDS-PAGE. Western blotting was carried out using the method of Towbin et al. [38]. Peroxidase-coupled secondary antibodies (Sigma) and the supersignal enhanced chemiluminescence system (Pierce, Rockford, IL, USA) were used.

### Embryo Multiple Tissue Northern Blot

The presence of Annexin A7 mRNA in mouse ES cells and embryos E7, E11, E15 and E17 was analyzed by northern blot analysis. An Embryo Multiple Tissue Northern Blot membrane was obtained from Clontech Laboratories (Heidelberg, FRG). It contained approximately 2 μg of poly(A)^+ ^RNA per lane from four different stages of mouse development. RNA from ES cells was isolated with the RNeasy kit (Qiagen). The membranes were hybridized with a random-primed [α-^32^P]-dATP-labelled full length annexin A7 cDNA probe and with a β-actin specific probe for control in ExpressHyb solution according to the manufacturer's directions.

## Authors' contributions

MR, SIRG, and CSC planned and carried out the experiments, evaluated the results, and drafted the manuscript. CH provided polyclonal anti-Annexin A7 antibodies and brain sections of the AnxA7 knock out mouse, and contributed to the analysis of data. DT provided the sections the human autopsy brain, analyzed them by immunofluorescence, and contributed to the analysis of data. AAN conceived of the studies and participated in its design. All authors read and approved the manuscript.

## References

[B1] Liemann S, Lewit-Bentley A (1995). Annexins: a novel family of calcium- and membrane-binding proteins in search of a function. Structure.

[B2] Raynal P, Pollard HB (1994). Annexins: the problem of assessing the biological role for a gene family. Biochem Biophys Acta.

[B3] Creutz CE, Pazoles CJ, Pollard HB (1978). Identification and purification of an adrenal medullary protein (synexin) that causes calcium-dependent aggregation of isolated chromaffin granules. J Biol Chem.

[B4] Kuijpers GA, Lee G, Pollard HB (1992). Immunolocalization of synexin (annexin VII) in adrenal chromaffin granules and chromaffin cells: evidence for a dynamic role in the secretory process. Cell Tissue Res.

[B5] Magendzo K, Shirvan A, Cultraro C, Srivastava M, Pollard HB, Burns AL (1991). Alternative splicing of human synexin mRNA in brain, cardiac, and skeletal muscle alters the unique N-terminal domain. J Biol Chem.

[B6] Selbert S, Fischer P, Pongratz D, Stewart M, Noegel AA (1995). Expression and localization of annexin VII (synexin) in muscle cells. J Cell Sci.

[B7] Clemen CS, Hofmann A, Zamparelli C, Noegel AA (1999). Expression and localisation of annexin VII (synexin) isoforms in differentiating myoblasts. J Muscle Res Cell Motil.

[B8] Herr C, Clemen CS, Lehnert G, Kutschkow R, Picker SM, Gathof BS, Zamparelli C, Schleicher M, Noegel AA (2003). Function, expression and localization of annexin A7 in platelets and red blood cells: Insights derived from an annexin A7 mutant mouse. BMC Biochemistry.

[B9] Clemen CS, Herr C, Hövelmeyer N, Noegel AA (2003). The lack of annexin A7 affects functions of primary astrocytes. Exp Cell Res.

[B10] Herr C, Smyth N, Ullrich S, Yun F, Sasse P, Hescheler J, Fleischmann B, Lasek K, Brixius K, Schwinger R, Fässler R, Schröder R, Noegel AA (2001). Loss of annexin A7 leads to alterations in frequency-induced shortening of isolated murine cardiomyocytes. Mol Cell Biol.

[B11] Zhang-Keck ZY, Burns AL, Pollard HB (1993). Mouse synexin (annexin VII) polymorphisms and a phylogenetic comparison with other synexins. Biochem J.

[B12] Olski TM, Noegel AA, Korenbaum E (2001). Parvin, a 42 kDa focal adhesion protein, related to the α-actinin superfamily. J Cell Sci.

[B13] Berry M, Gottlieb G (1974). Development of the cerebral neocortex of the rat. Aspects of neurogenesis.

[B14] Marotte LR, Leamey CA, Waite PME (1997). Timecourse of development of the Wallaby trigeminal pathway: III. Thalamocortical and corticothalamic projections. J Comp Neurol.

[B15] Hazelbag HM, van den Broek LJ, van Dorst EB, Offerhaus GJ, Fleuren GJ, Hogendoorn PC (1995). Immunostaining of chain-specific keratins on formalin-fixed, paraffin-embedded tissues: a comparison of various antigen retrieval systems using microwave heating and proteolytic pre-treatments. J Histochem Cytochem.

[B16] Kashima K, Yokoyama S, Daa T, Nakayama I, Nickerson PA, Noguchi S (1997). Cytoplasmic biotin-like activity interferes with immunohistochemical analysis of thyroid lesions: a comparison of antigen retrieval methods. Mod Pathol.

[B17] Kanai K, Nunoya T, Shibuya K, Nakamura T, Tajima M (1998). Variations in effectiveness of antigen retrieval pretreatments for diagnostic immunohistochemistry. Res Vet Sci.

[B18] Yuasa S (2001). Development of astrocytes in the mouse embryonic cerebrum tracked by tenascin-C gene expression. Arch Histol Cytol.

[B19] Clemen CS, Herr C, Lie AA, Noegel AA, Schröder R (2001). Annexin VII: an astroglial protein exhibiting a Ca^2+^-dependent subcellular distribution. NeuroReport.

[B20] Heizmann CW, Braun K (1992). Changes in Ca(2+)-binding proteins in human neurodegenerative disorders. Trends Neurosci.

[B21] Miller RJ (1995). Regulation of calcium homoeostasis in neurons: the role of calcium-binding proteins. Biochem Soc Trans.

[B22] Goda Y, Sudhof TC (1997). Calcium regulation of neurotransmitter release: reliably unreliable?. Curr Opin Cell Biol.

[B23] Verkhratsky AJ, Petersen OH (1998). Neuronal calcium stores. Cell Calcium.

[B24] Garcia ML, Strehler EE (1999). Plasma membrane calcium ATPases as critical regulators of calcium homeostasis during neuronal cell function. Front Biosci.

[B25] Mattson MP, Chan SL (2003). Neuronal and glial calcium signaling in Alzheimer's disease. Cell Calcium.

[B26] Mamiya N, Iino S, Mizutani A, Kobayashi S, Hidaka H (1994). Development-related and cell-type specific nuclear localization of annexin XI: immunolocalization analysis in rat tissues. Biochem Biophys Res Commun.

[B27] Zaks WJ, Creutz CE (1991). Ca(2+)-dependent annexin self-association on membrane surfaces. Biochemistry.

[B28] Brownawell AM, Creutz CE (1997). Calcium-dependent binding of sorcin to the N-terminal domain of synexin (annexin VII). J Biol Chem.

[B29] Mizutani A, Watanabe N, Kitao T, Tokumitsu H, Hidaka H (1995). The long amino-terminal tail domain of annexin XI is necessary for its nuclear localization. Arch Biochem Biophys.

[B30] Barwise JL, Walker JH (1996). Subcellular localization of annexin V in human foreskin fibroblasts: nuclear localization depends on growth state. FEBS Lett.

[B31] Barwise JL, Walker JH (1996). Annexins II, IV, V and VI relocate in response to rises in intracellular calcium in human foreskin fibroblasts. J Cell Sci.

[B32] Tomas A, Moss SE (2003). Calcium- and cell cycle-dependent association of annexin 11 with the nuclear envelope. J Biol Chem.

[B33] Eberhard DA, Karns LR, VandenBerg SR, Creutz CE (2001). Control of the nuclear-cytoplasmic partitioning of annexin II by a nuclear export signal and by p11 binding. J Cell Sci.

[B34] Jindal HK, Chaney WG, Anderson CW, Davis RG, Vishwanatha JK (1991). The protein-tyrosine kinase substrate, calpactin I heavy chain (p36), is part of the primer recognition protein complex that interacts with DNA polymerase alpha. J Biol Chem.

[B35] Vannahme C, Smyth N, Miosge N, Gösling S, Frie C, Paulsson M, Maurer P, Hartmann U (2002). Characterization of SMOC-1, a novel modular calcium-binding protein in basement membranes. J Biol Chem.

[B36] Langanger G, De Mey J, Adam H (1983). The sub-cellular localization of annexin V in cultured chick-embryo fibroblasts. Mikroskopie.

[B37] Adachi O, Kawai T, Takeda K, Matsumoto M, Tsutsui H, Sakagami M, Nakanishi K, Akira S (1998). Targeted disruption of the MyD88 gene results in loss of IL-1- and IL-18-mediated function. Immunity.

[B38] Towbin H, Staehelin T, Gordon J (1979). Electrophoretic transfer of proteins from polyacrylamide gels to nitrocellulose sheets: procedure and some applications. Proc Nat Acad Sci USA.

